# Adaptive Adjustments in Lower Limb Muscle Coordination during Single-Leg Landing Tasks in Latin Dancers

**DOI:** 10.3390/biomimetics9080489

**Published:** 2024-08-13

**Authors:** Xiangli Gao, Tianle Jie, Datao Xu, János Gál, Gusztáv Fekete, Minjun Liang, Yaodong Gu

**Affiliations:** 1 Faculty of Sports Science, Ningbo University, Ningbo 315211, China; 2 Faculty of Engineering, University of Pannonia, 8201 Veszprem, Hungary; 3 Department of Communication, Faculty of Electronics, Telecommunications and Information Technologies, Politehnica University of Timisoara, 300223 Timisoara, Romania; 4 Department of Material Science and Technology, AUDI Hungária Faculty of Vehicle Engineering, Széchenyi István University, 9026 Győr, Hungary; 5 Faculty of Engineering, University of Szeged, 6720 Szeged, Hungary

**Keywords:** dance biomechanics, muscle synergy, neuromuscular control patterns, adaptive adjustment, landing strategy

## Abstract

Previous research has primarily focused on evaluating the activity of individual muscles in dancers, often neglecting their synergistic interactions. Investigating the differences in lower limb muscle synergy during landing between dancers and healthy controls will contribute to a comprehensive understanding of their neuromuscular control patterns. This study enrolled 22 Latin dancers and 22 healthy participants, who performed a task involving landing from a 30 cm high platform. The data were collected using Vicon systems, force plates, and electromyography (EMG). The processed EMG data were subjected to non-negative matrix factorization (NNMF) for decomposition, followed by classification using K-means clustering algorithm and Pearson correlation coefficients. Three synergies were extracted for both Latin dancers and healthy participants. Synergy 1 showed increased contributions from the tibialis anterior (*p* < 0.001) and medial gastrocnemius (*p* = 0.024) in Latin dancers compared to healthy participants. Synergy 3 highlighted significantly greater contributions from the vastus lateralis in healthy participants compared to Latin dancers (*p* = 0.039). This study demonstrates that Latin dancers exhibit muscle synergies similar to those observed in healthy controls, revealing specific adjustments in the tibialis anterior and medial gastrocnemius muscles among dancers. This research illustrates how dancers optimize control strategies during landing tasks, offering a novel perspective for comprehensively understanding dancers’ neuromuscular control patterns.

## 1. Introduction

In the domains of biomechanics and neural control systems, the investigation into how dancers uphold equilibrium has remained a pivotal research pursuit [1,2,3,4,5]. It is widely known that dancers have superior balancing abilities compared to ordinary people [6,7,8]. This advantage is attributed to the particular demands of dance movements, including the rigorous requirements for posture control [1,9,10]. The lower limb biomechanical parameters of dancers undergo alterations through extensive and repetitive training [3,7,8]. Consequently, numerous researchers have begun to focus on this matter.

Nonetheless, the predominant approach in the current research for evaluating the lower limb stability of dancers is through traditional biomechanical parameters, such as kinematics, kinetics, and muscle activation [7,8]. For instance, dancers’ center of pressure and center of mass typically show less motion than non-dancers [8,11]. In the evaluation of muscle-related parameters, conventional electromyography is predominantly employed for the independent analysis of individual muscles [12,13]. In fact, a complete movement is not generated by independently controlling muscles, but rather requires the central nervous system to regulate the coordinated contraction of multiple muscles in order to accomplish it [13]. Therefore, traditional analytical methods may not adequately elucidate the coordinated role of multiple muscles in controlling movement.

The low-dimensional module constructed through the simplification of motor behavior is referred to as muscle synergy [14,15,16]. It deconstructs complex movements into simpler components, each executed by distinct muscle group. The contribution of each muscle in coordinated movement can be revealed when performing specific actions [17,18]. The presence of muscle synergy fosters the advancement of efficient and well-organized control [19,20]. The surface electromyography (EMG) signals obtained directly are unsuitable for the direct analysis of muscle synergy; instead, they necessitate the decomposition and extraction of muscle synergy patterns. This decomposition technique dissects the processed electromyography data into two low-dimensional components: muscle synergy vectors and activation coefficients [17]. The muscle synergy vectors delineate the relative weights of each muscle involved in synergy, while the activation coefficients delineate the relative role of each muscle in a specific task [21,22]. The non-negative matrix factorization (NNMF) posits the non-negativity of both muscle synergy vectors and activation coefficients. Through the multiplication of these vectors and coefficients within the matrix, EMG signals undergo reconstruction [23,24,25]. Previous studies required healthy participants to walk on a treadmill at speeds of 1, 2, 3, and 5 km per hour, with a safety harness supporting 35–95% of their body weight. Electromyographic data were recorded for the same-side limbs and trunk muscles. They identified five basic latent factors that account for about 90% of the total waveform variance in different muscles during gait. Ultimately, it was found that five component factors could explain a considerable portion of the total EMG pattern variance in muscles active during locomotion. This finding suggests that the activation of each muscle involves a dynamic weighting of these basic patterns. Previous research has elucidated that within particular contexts, muscle weights may undergo alterations contingent upon varying movement patterns or participant demographics [26,27]. Thus, comprehension of muscle synergy patterns serves to enhance the nuanced distribution of muscle weights.

The movements in Latin dance are often challenging, especially requiring high stability upon landing. Erroneous movements or suboptimal muscle coordination pose potential risks of injury [3,8]. An enhanced comprehension of the intricate muscle synergy patterns among Latin dancers facilitates their adept maintenance of bodily equilibrium, consequently fostering a profound insight into the mechanisms of control. Falling from a height poses a significant challenge to an individual’s balance, requiring coordination of the lower limbs to maintain stability [28,29,30]. This study observed landing movements using Vicon, force plates, and EMG, acquiring indicators related to muscle activation, kinematics, and dynamics. These indicators were useful for analyzing changes in the biomechanical parameters required by the lower limbs to maintain balance in an unstable state. Therefore, landing movements were the primary area of assessment in this study. Acting as a practical tool for evaluating balance proficiency, landing actions provide a unique insight into the intricate interplay between the neural mechanisms and motor responses crucial for maintaining stability [31,32]. Over time, dancers may experience changes in muscle synergy during landings. Our hypothesis suggests that Latin dancers demonstrate heightened levels of neuromuscular sophistication, potentially resulting in diverse distributions of muscle weights. For example, the frequent use of heel-raising maneuvers in Latin dance enhances ankle joint functionality, potentially leading dancers to preferentially employ ankle joint strategies to maintain bodily equilibrium. These findings are poised to enhance our understanding of dancers’ physiological responses and motor control mechanisms during the execution of complex movements.

Consequently, this study seeks to delve into the specific muscle adjustment strategies employed by Latin dancers during landing actions, thereby elucidating the central nervous system’s intricate regulation of muscular function. The implications of this research are profound, as it not only deepens our understanding of the complex mechanisms governing dancers’ corporeal control but also endeavors to enhance the stability of their movements. Building on the findings of this study, future research could potentially pivot towards the application of muscle regulation within the realm of tissue engineering. Moreover, unraveling the complexities of central nervous system-mediated muscle regulation is paramount for clinical practice, providing invaluable insights for the development of personalized rehabilitation protocols for individuals with balance disorders, including stroke, Parkinson’s disease, and a range of other movement disorders. Concurrently, investigations into muscle synergy are poised to propel advancements in the disciplines of biomechanics and motor control.

## 2. Materials and Methods

### 2.1. Participants

Sample sizes were calculated using G-Power software (version: 3.1.9.7; Henry University of Düsseldorf, Düsseldorf, Germany) [3,8]. A total of 22 Latin dancers (LD group; age 23.23 ± 3.21 years, height 1.65 ± 0.08 m, weight 52.34 ± 2.08 kg) and 22 participants from the healthy control group (CON group; age 21.76 ± 2.48 years, height 1.64 ± 0.07 m, weight 53.07 ± 2.23 kg) were recruited to participate in the study [24]. Initial screening and inclusion in the LD group was based on having more than 6 years of dance experience, maintaining a consistent dance routine, and engaging in at least 2 Latin dance training sessions per week; the CON maintained a regular exercise regimen, participating in physical activity for approximately one hour, two to three times per week. 

All participants enrolled in the experiment must have had no lower limb injuries within the past three months; otherwise, they would be deemed ineligible for participation. The purpose and procedures of the experiment were explained to all participants, who provided written consent. The experiment was approved by the Ethics Committee of Ningbo University. All subjects were informed of the research objectives, procedures, and signed written consent forms. This experiment received ethical approval from the Ethics Committee of Ningbo University (Approval Code: RAGH20240120).

### 2.2. Experimental Procedure

We placed 38 reflective markers on the subjects to capture their motion trajectories using a Vicon motion capture system (Oxford Metrics Ltd., Oxford, UK). The specific marker placements were as follows: shoulder peaks, external epicondyle of the humerus, medial epicondyle of the humerus, anterior superior iliac spine, radial styloid process, ulnar styloid process, upper outer thigh, mid-front thigh, lower outer thigh, external epicondyle of the femur, medial epicondyle of the femur, fibular head, upper outer calf, upper front side of calf, epicondyle of the fibula, medial tibial condyle, lateral midfoot, medial side of midfoot, fifth metatarsophalangeal joint, first metatarsophalangeal joint, toes, toes, bilateral middle posterior superior iliac spine, bilateral middle posterior superior iliac spine [31,33]; the subjects’ kinetic data were recorded via an AMTI force plate (Watertown, MA, USA). The sampling frequencies for kinetics and kinematics were 1000 Hz and 200 Hz, respectively [12,34]. 

**Figure 1:**
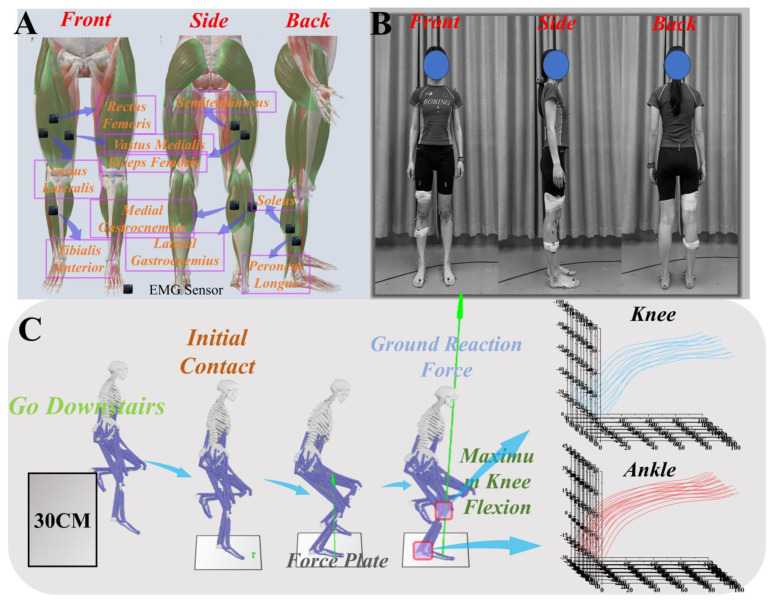
Illustration of (**A**) the EMG acquisition illustration; (**B**) the musculoskeletal model; and (**C**) the motion capture process.

We used sixteen-channel wireless EMG (Delsys, Boston, MA, USA) to collect relevant data around the lower limb muscles of the participants, with a sampling frequency of 1000 Hz [12]. During the acquisition of EMG data, we meticulously identified the anatomical origin and insertion points of each muscle through both palpation and consultation of detailed anatomical diagrams, specifically detailed as follows: Semitendinosus (ST): the origin is precisely located at the ischial tuberosity, while the insertion is at the superior aspect of the medial condyle of the tibia. Biceps Femoris (BF): originates from the ischial tuberosity and inserts on the lateral side of the fibular head. Vastus Medialis (VM): the origin is situated at the medial condyle of the femur, with insertion at the medial condyle of the tibia. Rectus Femoris (RF): begins at the anterior inferior iliac spine of the hip joint and inserts at the tibial tuberosity. Vastus Lateralis (VL): originates from the greater trochanter of the femur and inserts along the lateral border of the patella and the lateral condyle of the tibia. Tibialis Anterior (TA): starts on the anterior lateral surface of the tibia, and inserts at the medial base of the first metatarsal and the medial cuneiform. Soleus (SOL): originates from the posterior lower parts of the tibia and fibula and inserts at the upper region of the Achilles tendon. Gastrocnemius Medial (GM): the origin is at the medial condyle of the femur, with insertion at the upper segment of the Achilles tendon. Gastrocnemius Lateral (GL): begins at the lateral condyle of the femur and inserts at the superior part of the Achilles tendon. Peroneus Longus (PL): originates from the upper lateral side of the fibula, and inserts at the base of the first metatarsal and the medial cuneiform. To ensure optimal sensor placement accuracy, we carefully marked the origin and insertion points on the skin and positioned the sensors at the midpoint of each muscle. This strategic placement was intended to ensure the precise and effective capture of muscle-related electrical activity data. (Figure 1A shows the specific placement locations of the EMG sensors). 

Maximum Voluntary Contractions (MVCs) were also recorded for these 10 muscle groups to standardize muscle activation. Subjects were assigned to either a sitting or standing posture according to the specific muscles under assessment. The sitting posture was utilized for evaluating the anterior and medial thigh muscles, providing optimal stability and support for accurate measurement. In contrast, the standing posture was employed for assessing the calf muscles, as it more closely simulated their functional state during actual activities. This strategic selection of postures was designed to enhance the precision of the measurements. Participants were instructed by the researcher to exert maximal effort in contracting the target muscles for 3 s, followed by a 10 s rest period. Each test was performed in multiple repetitions, with peak EMG signal values recorded for each trial. 

Before the experiment, participants were required to wear tight-fitting shorts and short-sleeved shirts. The researchers then prepared the skin by cleaning it with alcohol and used a medical razor to remove hair from the lower limb areas to be sampled, enhancing the conductivity of the electromyographic signals [23]. To prevent injury and ensure accurate data collection, participants performed a warm-up routine, which included a 10-min jog at a pace of 8 km/h [35], followed by 5 min of full-body stretching exercises. Finally, to familiarize participants with the experimental procedures and movements, they practiced the experimental actions approximately three times.

The subjects are initially required to assume a standard anatomical position for the collection of a series of static data. Throughout the entirety of the experimental procedure, a supervisor will be appointed to oversee proceedings [8]. Under the supervision of the appointed supervisor, participants will execute a landing maneuver from a platform elevated 30 cm above the ground. During the landing, participants will utilize their dominant leg, typically the one they employ for kicking a football. Following the landing, participants will maintain single-leg support for three seconds until deemed fully stable, marking successful completion. The landing action will take place on the force plate (AMTI) as depicted in Figure 1C. All actions will be performed barefoot. Note: During the experiment, participants might instinctively lower their supporting leg to test the height of the platform before landing, which could potentially reduce the platform height and affect the experimental results. Therefore, participants had to ensure that the height of their supporting leg matched the height of the platform during the landing action.

### 2.3. Data Processing

We defined initial contact as a ground reaction force exceeding 10 N [8,36]. The Vicon Nexus 2.16 software was used to export data in c3d format, capturing the participants’ kinematic and kinetic data. MATLAB R2022b was employed for subsequent data processing, which included coordinate transformation, low-pass filtering, format conversion, and data extraction [12]. We transformed the coordinate systems of the kinematic and kinetic data to coordinates suitable for OpenSim simulation modeling. We filtered the kinematic and kinetic data using cutoff frequencies of 10 Hz and 20 Hz, respectively, and then converted the extracted kinematic and kinetic data into TRC (marker trajectories) and MOT (force plate data) formats for subsequent processing using OpenSim (Stanford University, Stanford, CA, USA). Within OpenSim, scaling tools were utilized to create personalized body measurement models for each participant, incorporating limb lengths to determine muscle origin and insertion points. The software’s inverse kinematics (IK) tool computed joint angles during the stance phase of kicking movements, resulting in motion files (mot). Following this, the residual reduction algorithm (RRA) was applied to refine kinematic data, improving the dynamic data accuracy to match measured data.

**Figure 2:**
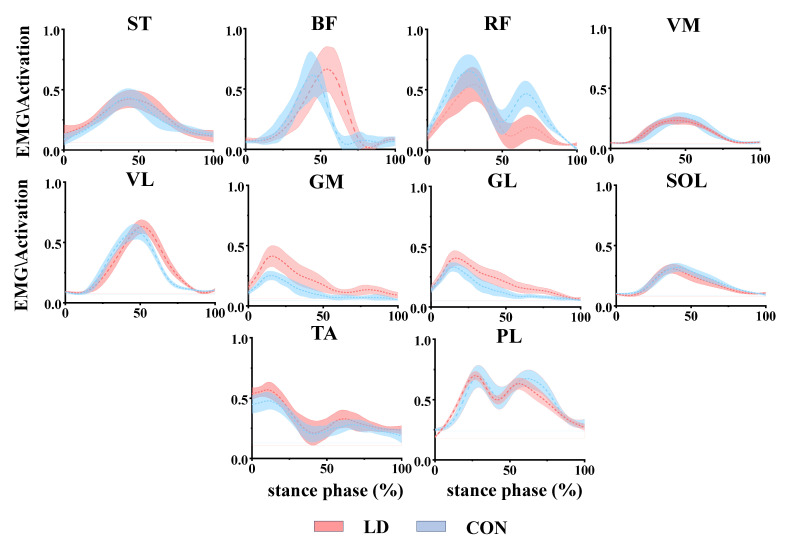
Normalized muscle activity (mean ± SD) in Latin dancers (LD) and healthy control individuals (CON) during the landing task. The red area represents LD, and the blue area represents CON. ST: semitendinosus; BF: biceps femoris; RF: rectus femoris; VL: vastus lateralis; VM: vastus medialis; GM: medial gastrocnemius; SOL: soleus; GL: gastrocnemius lateralis; TA: tibialis anterior; PL: peroneus longus.

Before performing full-wave rectification, the EMG data underwent a 4th-order band-pass filtering between 10 and 500 Hz, followed by smoothing using a 10 Hz low-pass filter [12]. Subsequently, signal normalization was performed by scaling the smooth activation data of each muscle to its maximum observed activation across all trials, and standardizing time to 101 data points. The following shows the EMG activation of ten muscles during the landing phase between LD and CON (Figure 2) [23]. 

### 2.4. Extraction of Muscle Synergies

**Figure 3:**
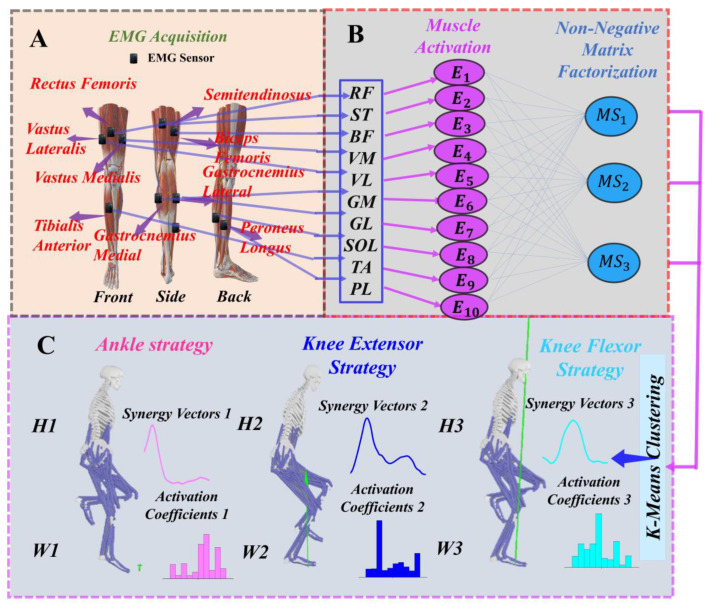
(**A**) Illustration of the specific locations of EMG sensors. (**B**) Non-negative matrix factorization workflow. (**C**) Illustration of the module of the movement strategy. ST: semitendinosus; BF: semitendinosus; RF: semitendinosus; VM: vastus medialis; VL: vastus lateralis; GM: vastus lateralis; GL: gastrocnemius lateralis; SOL: soleus; TA: tibialis anterior; PL: tibialis anterior.

Using the NNMF algorithm to extract the muscle synergies of the subjects [17,23,24]. To extract muscle synergy effects with equal weighting, each muscle’s activation is divided by its standard deviation to achieve unit variance [17]. The EMG data matrix E is organized as m × n×trial repetitions, where m denotes the number of muscles and n represents the number of sampled data points [37,38,39]. The processed electromyographic data are inputted into the NNMF algorithm. Decomposing the data into two linear combinations: synergy vectors and activation coefficients. Here, N represents the number of synergies, where $W_{i}$ denotes the weight vector, which indicates the relative contribution of individual muscles in organizing the corresponding motor modules, while $H_{i}$ signifies the activation coefficients, reflecting the changes in the temporal order of the synergy vectors $W_{i}$ modulated by the nervous system. ε denotes an error term, and NNMF allows for some reconstruction errors (The specific process of muscle synergy decomposition is shown in Figure 3) [17].

(1) $$E_{\left(m\times n\right)}=\sum_{i=1}^{N}W_{\left(m\times k\right)}\times H_{\left(k\times n\right)}+\varepsilon_{\left(m\times n\right)}$$

Subsequently, as the number of muscle synergies increases, the NNMF algorithm is iteratively applied. For instance, in a scenario with one synergy, the algorithm would extract one set of synergy vectors and one activation coefficient [13]. 

Using Variance Accounted For (VAF) to determine the number of muscle synergies, the number of synergies needs to satisfy the criteria that the total variance accounted for ${VAF}_{Total}$ ≥ 90%, and that the variance accounted for by each muscle ${VAF}_{{Each}_{m}}$ ≥ 75% [40,41].

(2) $${VAF}_{Total}=1-\frac{\sum_{i=1}^{p}\sum_{j=1}^{n}{\left(\varepsilon_{i,j}\right)}^{2}}{\sum_{i=1}^{p}\sum_{j=1}^{n}{\left(E_{i,j}\right)}^{2}}$$

p represents the number of muscles, n represents the number of time points, and m represents each muscle [13].

(3) $${VAF}_{{Each}_{m}}=1-\frac{\sum_{j=1}^{n}{\left(\varepsilon_{m,j}\right)}^{2}}{\sum_{j=1}^{n}{\left(E_{m,j}\right)}^{2}}$$

To identify synergistic effects, we employed an unsupervised method based on k-means clustering to classify them, aiming to minimize potential operator-dependent biases in the classification [17]. This involves clustering multiple clusters ranging from 1 to the number of muscles, and then clustering the synergy patterns based on the number of centroids obtained.

### 2.5. Statistical Analysis

Before conducting statistical analysis, the Shapiro–Wilk normality test is performed on the dataset to assess whether the data conform to a normal distribution [34]. Subsequently, an independent *t*-test is employed to carefully examine the differences between the two groups. To comprehensively assess the lower limb movement patterns of Latin dancers, within the context of statistical parametric mapping (SPM) analysis, we extracted the entire dataset and used a custom MATLAB script to expand the data from posture phases into a time-series curve consisting of 101 data points [42]. Subsequently, we employed the SPM 1d independent sample test script for statistical analysis, aiming to observe changes in the lower limb muscle activation coefficients and joints throughout the entire movement process [43,44]. 

To compare the similarity of muscle synergy vectors, calculate the Pearson correlation coefficient r. This is achieved by measuring the covariance between the two variables to assess their linear relationship, then normalizing the covariance by their respective standard deviations to obtain a value between −1 and 1, representing the strength and direction of the correlation between the two variables.

(4) $$r=\frac{\sum (x_{i}-\bar{x})(y_{i}-\bar{y})}{\sqrt{\sum (x_{i}-\bar{x}{)}^{2}\sum (y_{i}-\bar{y}{)}^{2}}}$$

The variable r denotes the Pearson correlation coefficient, where $x_{i}$ and $y_{i}$ are the observed values of the two variables, and $\bar{x}$ and $\bar{y}$ are their respective means. The classification of r values is as follows: 0.7 to 1.0 indicates strong correlation; 0.3 to 0.7 indicates moderate correlation; and less than 0.3 indicates weak correlation. Therefore, should r > 0.6, we deem the two synergies akin to each other and amalgamate them accordingly.

## 3. Results

### 3.1. Kinematics and Kinetics

**Figure 4:**
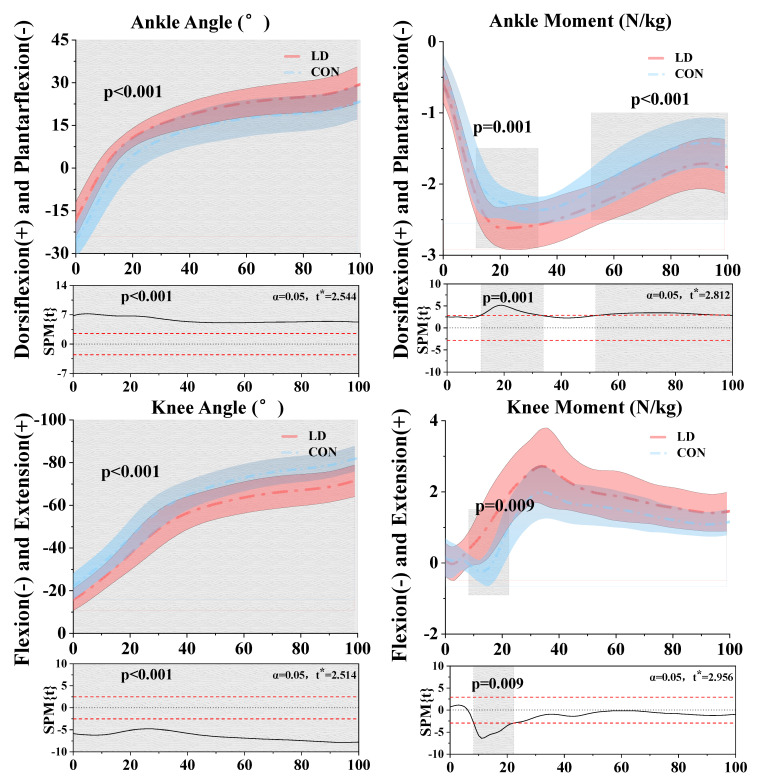
Illustration of the results between LD and CON, with the lower limb showing the statistical parametric mapping outputs for the ankle angle and moment; and knee angle and moment during the stance phase. The values of t* are shown on the left of each image. Grey shades represent the significant differences and t-values of the SPM or all participants, dashed red lines represent the results at *p* = 0.05.

In this study, the Shapiro–Wilk test was performed, yielding a *p*-value greater than 0.05. This result suggests that the null hypothesis cannot be rejected, thereby allowing the data to be regarded as approximately normally distributed. SPM analysis for the ankle joint results indicated that LD exhibited a notably higher dorsiflexion angle compared to CON throughout the 0–100% stance phase (*p* < 0.001). Moreover, LD demonstrated a significantly greater moment than CON at 11.52–33.32% (*p* = 0.001) and 52.07–100% (*p* < 0.001) of the stance phases. SPM analysis for the knee joint showed that LD exhibited a significantly greater extension angle than CON throughout the 0–100% stance phase (*p* < 0.001) and a notably higher moment than CON during the 8.23–22.34% stance phases (*p* = 0.009) (Figure 4).

### 3.2. Selection of the Number of Synergies

**Figure 5:**
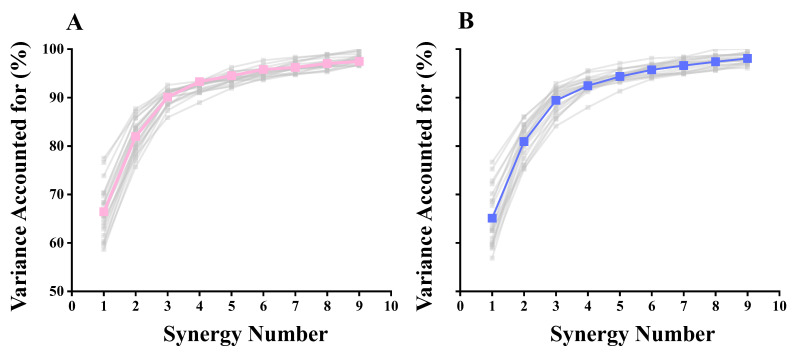
The variance accounted for, measured for the synergies. The pink and blue lines represent the average. (**A**) the LD group; (**B**) the CON group.

After k-means clustering, the results revealed no difference in the number of synergies between the LD and CON groups during the landing task (Table 1). Furthermore, we identified 2–5 synergies. In most cases, three synergies are sufficient to adequately reconstruct the original electromyographic signals. Therefore, we established three fundamental synergies (Figure 4), abbreviated as S1, S2, and S3 (Figure 5).

### 3.3. Similarity of Muscle Synergies

**Figure 6:**
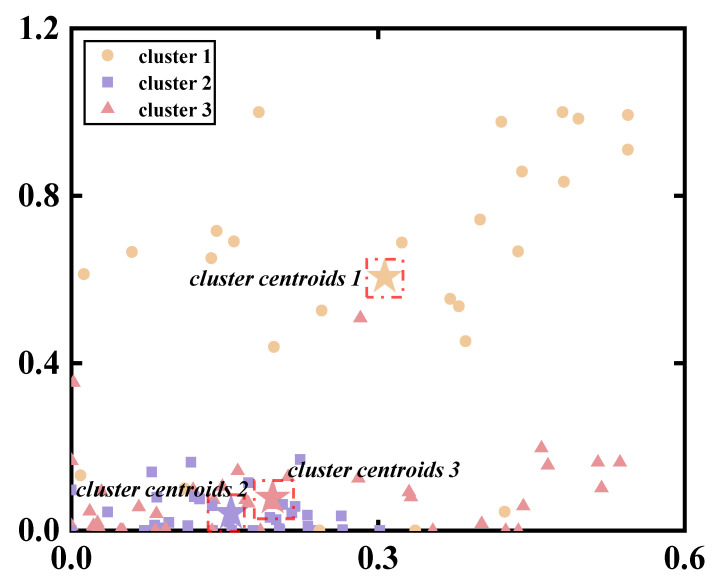
Visualizations of K-means clustering results for all synergy vectors in the CON group.

By using the Silhouette coefficient to evaluate the clustering effect, we found that when i = 3, the clustering effect was optimal. Three clustering centers were used for the reference synergy (Figure 6). 

**Figure 7:**
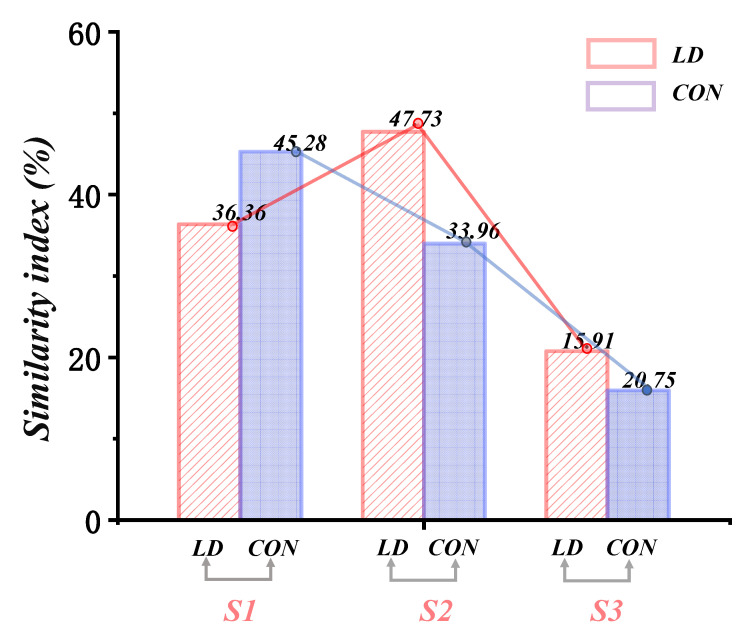
The correlation indices of synergy vectors and reference synergies between LD and CON. S1: muscle synergy 1; S2: muscle synergy 2; S3: muscle synergy 3.

The synergy patterns were classified using the Pearson correlation coefficient. Subsequently, it was observed that synergy patterns 1 (LD: 45.28%; CON: 36.36%) and 2 (LD: 33.96%; CON: 47.72%) exhibited higher degrees of similarity in comparison to synergy pattern 3 (LD: 15.90%; CON: 20.75%) (Figure 7).

### 3.4. Functional Interpretation of Muscle Synergies

The muscle is engaged once its weight surpasses the threshold of 0.3.

For synergy 1, activation coefficient 1 primarily reflects the initial contact phase. Considering the muscle weights from synergy vector 1, it predominantly drives ankle strategy, functionally related to landing preparation.

For synergy 2, activation coefficient 2 primarily reflects the necessity of sustaining one’s own weight post-initial contact. Upon consideration of the muscle weights derived from synergy vector 2, it predominantly illustrates a knee extensor strategy dominated by the rectus femoris.

**Figure 8:**
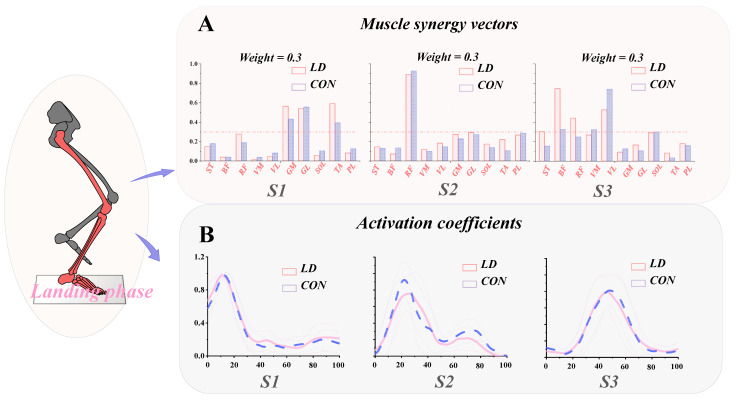
(**A**) Muscle synergy vectors (and muscle-specific weightings) extracted from each group during the landing task. (**B**) Activation coefficients extracted from each group during each task. S1: muscle synergy 1; S2: muscle synergy 2; S3: muscle synergy 3.

For synergy 3, activation coefficient 3 indicates the phase of maintaining balance after completing the landing action. Considering the muscle weights from synergy vector 3, it primarily demonstrates a knee strategy dominated by the BF and VL muscles (Figure 8).

### 3.5. General Characteristics of Muscle Synergies

From the results, in synergy 1, the relative weights of LD’s GM and TA muscles were greater than CON (*p* = 0.024, *p* < 0.001), while LD’s VM and SOL muscles’ relative weights were smaller than CON (*p* = 0.001, *p* = 0.002). There were no significant differences in the relative weights of other muscles.

In synergy 2, there were no significant differences in the relative weights of the muscles. 

In synergy 3, except for LD’s VL muscle weight being smaller than CON (*p* = 0.039), the relative weights of the ST and BF muscles were greater than CON (*p* = 0.046, *p* < 0.001), and there were no significant differences in the relative weights of the remaining muscles (Table 2).

## 4. Discussion

The present study pioneers an examination of the neurological control strategies utilized by Latin dancers, focusing on their muscle synergy. This investigation is of paramount significance, as it introduces a novel approach to understanding how the nervous system regulates muscular activity during movement execution. Consequently, the primary aim of our research is to delve into the intricate adjustments of muscle synergy observed in LD during landing maneuvers, with the overarching goal of maintaining bodily equilibrium. The study findings suggest a similarity in the expression of synergy effects between LD and CON. However, while no significant differences were observed in synergy 2 between the two groups, distinct individual variations were evident in both synergy 1 and synergy 3. This underscores how individual differences across various types of muscle synergy might influence motor control. In synergy 1, LD exhibit higher weights of the TA and GM compared to CON; whereas in synergy 3, the LD group demonstrates greater weights of the BF than the CON group, while the VL exhibits lower weights than non-dancers. This outcome substantiates our hypothesis: the neural control system of Latin dancers adjusts muscle strategies with prolonged practice, showing a predisposition towards employing ankle joint strategies. Through an understanding of muscle synergy patterns, we aspire to offer novel insights into the movement patterns of dancers.

Our research findings indicate that there were no significant differences in the quantity of muscle synergy between LD and healthy participants, CON. This observation may suggest that they employ similar neuromuscular control strategies during landing actions, aligning with previous research outcomes [13,17]. Consequently, our focus shifted towards examining the weights of muscle synergy. In synergy 1, as reflected primarily by activation coefficients during the initial contact phase of landing, the TA, GM, and GL were identified as the major contributing muscles. These muscles, predominantly surrounding the ankle joint, directly engage in ankle dorsiflexion and plantarflexion movements [45,46]. When individuals encounter challenges to their equilibrium, the nervous system adapts and triggers the activation of the muscles encompassing the ankle joint, whose vigorous contractions regulate the motion of the ankle joint [47,48,49]. The TA is one of the primary dorsiflexors of the ankle joint [47]. Its activation expands the range of motion of the ankle joint, possibly contributing to the greater dorsiflexion angle observed in LD compared to CON during the stance phase (Figure 4). Within a certain range, an increase in ankle dorsiflexion can reduce the risk of ankle sprains and enhance ankle stability, as it renders the joint more adept at supporting the body’s weight and absorbing forces from movement or external pressure [50]. By observing Figure 8, we discern a predominantly ankle-driven strategy, wherein the musculature surrounding the ankle joint is mobilized to maintain equilibrium, possibly in response to postural adjustments [51,52,53].

In synergy 2, no significant differences were observed between LD and CON. This suggests that both groups employ similar strategies during the phase of initial weight-bearing following contact. Subsequently, the activation of the RF assists in controlling knee extension to support the body and stabilize posture during landing [54]. This muscular activation serves to alleviate the burden on other joints and muscles of the lower limbs while maintaining balance and stability. Despite the activation of the rectus femoris in both groups, the activation coefficients did not exhibit significant differences, indicating the preservation of synergy patterns in Latin dancers during the weight-bearing phase.

In synergy 3, the orchestration of muscle activity around the knee joint assumes paramount importance in preserving individual equilibrium post landing [55,56,57,58,59]. As depicted in Figure 8, the knee joint strategy is predominantly governed by the BF and VL. Remarkably, individuals in the CON cohort demonstrate a preference for relying on the VL. This inclination likely arises from the initial landing phase, where the contribution of ankle joint muscles in CON may prove insufficient to adequately stabilize the body. Consequently, there is a notable upsurge in the activation of knee joint muscles to enhance balance maintenance [60]. The vastus lateralis, responsible for knee extension movements, emerges as a pivotal contributor in this endeavor [61,62]. Interestingly, observations reveal that CON exhibits a higher ankle dorsiflexion angle compared to LD. This observation suggests CON’s proficiency in maintaining bodily stability through nuanced adjustments in the sagittal plane movement range of the knee joint. The amplified angle of knee extension prompts heightened muscle activation around the joint, notably augmenting the contribution of the vastus lateralis. Such muscular adaptation compensates for the comparatively modest muscle engagement around the ankle joint, thereby fortifying overall stability [55].

This study unveils the muscular synergy of Latin dancers, with implications for the advancement of disciplines such as motor control and dance studies. Nevertheless, we acknowledge the following several limitations within our research: (1) limited studies exist on the muscular synergy of LD, thus restricting the depth of our analysis; (2) while our findings reveal no significant differences between LD and CON in synergy 2, we cannot entirely rule out other potential influencing factors, such as gender. Male and female dancers may employ distinct muscle activation patterns and coordination strategies when performing the same movements, leading to variations in neuromuscular control. Furthermore, differences in muscle strength between genders can influence performance and stability during landing [63]. Incorporating male dancers into the study would enable a more comprehensive analysis of landing strategies and provide deeper insights into the neuromuscular control patterns specific to Latin dancers. (3) The definition of the dominant leg in this study may overlook individual differences in leg strength, flexibility, or other biomechanical factors, potentially affecting the accuracy of measurements and the reliability of results. Additionally, variations in dominant legs among different subjects could contribute to variability in the research outcomes. (4) Our study focuses solely on specific landing phases, whereas dance performances may encompass more intricate and diversified movements and techniques. Consequently, our conclusions may not comprehensively encompass the muscular synergy patterns of LD across different dance actions. To comprehensively explore the muscular synergy of LD, future research could consider enlarging sample sizes, establishing more precise inclusion criteria for participants, incorporating additional dance technical parameters, utilizing more sophisticated measurement tools, and conducting long-term observational studies to more accurately elucidate the muscular synergy of LD.

## 5. Conclusions

While Latin dancers frequently execute intricate movements, they exhibit no significant disparity in muscle synergy quantities during the landing process compared to healthy counterparts. However, through an analysis of the activation coefficients, the study reveals how dancers optimize their control strategies during landing, including specific adjustments of the tibialis anterior and medial gastrocnemius muscles. This movement pattern serves to stabilize foot positioning and mitigate instability during landing. By delving into the muscle synergy patterns of Latin dancers, we deepen our comprehension of dancer equilibrium, laying the groundwork for future endeavors towards achieving efficient and orchestrated control. Additionally, this study reveals that, despite the heightened activation of the TA and GM during landing, the activation levels of specific knee joint muscles in Latin dancers remain marginally lower compared to non-dancers. This disparity has not resulted in a substantial enhancement in landing control and stability. Given the pivotal role of the knee joint in preserving overall body equilibrium, it is advisable for dancers and Latin dance instructors to integrate specialized exercises aimed at improving knee joint stability into their training regimens. Such an approach is likely to bolster landing control and stability, thereby refining overall performance.

## Figures and Tables

**Table 1:** Descriptive data on the number of muscle synergies, global VAF, and local VAFs for each group.

	LD Mean (SD)	CON Mean (SD)	*p*-Value
$N_{syn}$	3.43 (0.51)	3.31 (0.57)	0.506
Global VAF (%)			
With two synergies	81.03 (3.45)	81.94 (3.47)	0.393
With three synergies	93.61 (2.57)	90.41 (2.56)	0.303
With four synergies	92.55 (1.62)	92.31 (1.12)	0.581
Local VAFs with three synergies (%)			
ST	88.91 (5.42)	90.41 (5.97)	0.394
BF	90.50 (4.85)	91.41 (4.57)	0.527
RF	94.33 (4.32)	96.36 (2.10)	0.056
VM	89.93 (5.19)	89.72 (4.49)	0.886
VL	92.66 (3.53)	94.45 (3.25)	0.092
GM	91.56 (3.46)	92.20 (2.58)	0.496
GL	91.47 (3.28)	93.02 (1.53)	0.059
SOL	92.51 (3.78)	92.46 (3.14)	0.964
TA	91.48 (2.65)	89.71 (3.41)	0.065
PL	92.93 (2.44)	91.32 (4.08)	0.122

Abbreviations. ST: semitendinosus; BF: semitendinosus; RF: semitendinosus; VM: vastus medialis; VL: vastus lateralis; GM: vastus lateralis; GL: gastrocnemius lateralis; SOL: soleus; TA: tibialis anterior; PL: tibialis anterior.

**Table 2:** Comparison of muscle synergy vectors during landing between the LD and CON groups.

Synergy Vectors	Muscle Synergy 1			Muscle Synergy 2			Muscle Synergy 3		
Mean (SD)	LD	CON	*p*-Value	LD	CON	*p*-Value	LD	CON	*p*-Value
ST	0.15 (0.08)	0.18 (0.11)	0.288	0.14 (0.12)	0.14 (0.18)	0.848	0.30 (0.18)	0.15 (0.17)	0.046 *
BF	0.04 (0.06)	0.04 (0.06)	0.946	0.07 (0.13)	0.12 (0.13)	0.31	0.75 (0.20)	0.32 (0.21)	<0.001 *
RF	0.28 (0.29)	0.19 (0.24)	0.291	0.84 (0.28)	0.91 (0.15)	0.283	0.44 (0.31)	0.25 (0.16)	0.076
VM	0.01 (0.02)	0.04 (0.04)	0.010 *	0.12 (0.11)	0.09 (0.08)	0.329	0.27 (0.12)	0.32 (0.09)	0.204
VL	0.04 (0.04)	0.08 (0.16)	0.282	0.19 (0.23)	0.13 (0.14)	0.372	0.53 (0.27)	0.74 (0.20)	0.039 *
GM	0.56 (0.20)	0.43 (0.16)	0.024 *	0.23 (0.23)	0.26 (0.17)	0.643	0.09 (0.08)	0.13 (0.07)	0.307
GL	0.54 (0.15)	0.56 (0.18)	0.771	0.26 (0.17)	0.29 (0.17)	0.549	0.17 (0.10)	0.11 (0.08)	0.119
SOL	0.06 (0.04)	0.10 (0.06)	0.002 *	0.17 (0.11)	0.13 (0.08)	0.211	0.29 (0.10)	0.30 (0.05)	0.797
TA	0.59 (0.19)	0.39 (0.12)	<0.001 *	0.20 (0.15)	0.13 (0.16)	0.112	0.08 (0.08)	0.03 (0.03)	0.093
PL	0.08 (0.11)	0.13 (0.15)	0.298	0.25 (0.16)	0.29 (0.18)	0.476	0.18 (0.15)	0.16 (0.08)	0.683

Note. * indicates a significant difference between LD and CON during the stance phase (*p* < 0.05).

## Data Availability

The data that support the findings of this study are available upon reasonable request from the corresponding author. The data are not publicly available due to privacy or ethical restrictions.

## References

[1] Aquino J., Amasay T., Shapiro S., Kuo Y.-T., Ambegaonkar J.P. (2021). Lower extremity biomechanics and muscle activity differ between ‘new’ and ‘dead’ pointe shoes in professional ballet dancers. Sports Biomech..

[2] Wang Y., Watanabe K., Asaka T. (2019). Effect of dance on multi-muscle synergies in older adults: A cross-sectional study. BMC Geriatr..

[3] Gao X., Xu D., Li F., Baker J.S., Li J., Gu Y. (2023). Biomechanical Analysis of Latin Dancers’ Lower Limb during Normal Walking. Bioengineering.

[4] Hansberger B.L., Acocello S., Slater L.V., Hart J.M., Ambegaonkar J.P. (2018). Peak lower extremity landing kinematics in dancers and nondancers. J. Athl. Train..

[5] Koh K., Park Y.S., Park D.W., Shim J.K. (2020). Dance training improves the CNS’s ability to utilize the redundant degrees of freedom of the whole body. Sci. Rep..

[6] Liu Y.-T., Lin A.-C., Chen S.-F., Shih C.-J., Kuo T.-Y., Wang F.-C., Lee P.-H., Lee A.P. (2022). Superior gait performance and balance ability in Latin dancers. Front. Med..

[7] Kiliç M., Nalbant S.S. (2022). The effect of latin dance on dynamic balance. Gait Posture.

[8] Fotaki A., Triantafyllou A., Koulouvaris P., Skouras A.Z., Stasinopoulos D., Gkrilias P., Kyriakidou M., Stasi S., Antonakis-Karamintzas D., Tsolakis C.J.S. (2024). Excessive Knee Internal Rotation during Grand Plié in Classical Ballet Female Dancers. Sports.

[9] Janura M., Procházková M., Svoboda Z., Bizovská L., Jandová S., Konečný P. (2019). Standing balance of professional ballet dancers and non-dancers under different conditions. PLoS ONE.

[10] Azevedo A.M., Oliveira R., Vaz J.R., Cortes N. (2020). Oxford foot model kinematics in landings: A comparison between professional dancers and non-dancers. J. Sci. Med. Sport.

[11] Krityakiarana W., Jongkamonwiwat N. (2016). Comparison of balance performance between Thai classical dancers and non-dancers. J. Dance Med. Sci..

[12] Kovács B., Csala D., Sebestyén Ö., Matlák J., Groszmann Á., Tihanyi J., Petridis L.J. (2023). Arm swing during vertical jumps does not increase EMG activity of the lower limb muscles. Phys. Act. Health.

[13] Kim H., Palmieri-Smith R., Kipp K. (2023). Muscle synergies in people with chronic ankle instability during anticipated and unanticipated landing-cutting tasks. J. Athl. Train..

[14] Ivanenko Y.P., Grasso R., Zago M., Molinari M., Scivoletto G., Castellano V., Macellari V., Lacquaniti F. (2003). Temporal components of the motor patterns expressed by the human spinal cord reflect foot kinematics. J. Neurophysiol..

[15] Ting L.H., McKay J.L. (2007). Neuromechanics of muscle synergies for posture and movement. Curr. Opin. Neurobiol..

[16] d’Avella A., Bizzi E. (2005). Shared and specific muscle synergies in natural motor behaviors. Proc. Natl. Acad. Sci. USA.

[17] Munoz-Martel V., Santuz A., Bohm S., Arampatzis A. (2021). Proactive modulation in the spatiotemporal structure of muscle synergies minimizes reactive responses in perturbed landings. Front. Bioeng. Biotechnol..

[18] Torricelli D., Barroso F., Coscia M., Alessandro C., Lunardini F., Bravo Esteban E., d’Avella A. (2016). Muscle synergies in clinical practice: Theoretical and practical implications. Emerg. Ther. Neurorehabil..

[19] Tresch M.C., Saltiel P., Bizzi E. (1999). The construction of movement by the spinal cord. Nat. Neurosci..

[20] d’Avella A., Saltiel P., Bizzi E. (2003). Combinations of muscle synergies in the construction of a natural motor behavior. Nat. Neurosci..

[21] Torres-Oviedo G., Ting L.H. (2007). Muscle synergies characterizing human postural responses. J. Neurophysiol..

[22] Hug F. (2011). Can muscle coordination be precisely studied by surface electromyography?. J. Electromyogr. Kinesiol..

[23] Chang H.B., Cen X.Z. (2024). Can running technique modification benefit patellofemoral pain improvement in runners? A systematic review and meta-analysis. Int. J. Biomed. Eng. Technol..

[24] Xu D., Zhou H., Quan W., Gusztav F., Baker J.S., Gu Y. (2023). Adaptive neuro-fuzzy inference system model driven by the non-negative matrix factorization-extracted muscle synergy patterns to estimate lower limb joint movements. Comput. Methods Programs Biomed..

[25] Jie T., Xu D., Zhang Z., Teo E.-C., Baker J.S., Zhou H., Gu Y. (2024). Structural and Organizational Strategies of Locomotor Modules during Landing in Patients with Chronic Ankle Instability. Bioengineering.

[26] Ivanenko Y.P., Poppele R.E., Lacquaniti F. (2004). Five basic muscle activation patterns account for muscle activity during human locomotion. J. Physiol..

[27] Cappellini G., Ivanenko Y.P., Poppele R.E., Lacquaniti F. (2006). Motor patterns in human walking and running. J. Neurophysiol..

[28] Abt J.P., Sell T.C., Laudner K.G., McCrory J.L., Loucks T.L., Berga S.L., Lephart S.M. (2007). Neuromuscular and biomechanical characteristics do not vary across the menstrual cycle. Knee Surg. Sports Traumatol. Arthrosc..

[29] Ageberg E., Roberts D., Holmström E., Fridén T. (2005). Balance in single-limb stance in patients with anterior cruciate ligament injury: Relation to knee laxity, proprioception, muscle strength, and subjective function. Am. J. Sports Med..

[30] Sell T.C., Ferris C.M., Abt J.P., Tsai Y.S., Myers J.B., Fu F.H., Lephart S.M. (2007). Predictors of proximal tibia anterior shear force during a vertical stop-jump. J. Orthop. Res..

[31] Xu D., Zhou H., Quan W., Ma X., Chon T.-E., Fernandez J., Gusztav F., Kovács A., Baker J.S., Gu Y. (2024). New insights optimize landing strategies to reduce lower limb injury risk. Cyborg Bionic. Syst..

[32] Xu D., Zhou H., Quan W., Jiang X., Liang M., Li S., Ugbolue U.C., Baker J.S., Gusztav F., Ma X. (2024). A new method proposed for realizing human gait pattern recognition: Inspirations for the application of sports and clinical gait analysis. Gait Posture.

[33] Zhou H., Ugbolue U.C. (2024). Biomechanical Analysis of Lower Limbs Based on Unstable Condition Sports Footwear: A Systematic Review. Phys. Act. Health.

[34] Pan J.W., Ho M.Y.M., Loh R.B.C., Iskandar M.N.S., Kong P. (2023). Foot morphology and running gait pattern between the left and right limbs in recreational runners. Phys. Act. Health.

[35] Kang Z.H., Jiang X.Y. (2024). The effect of running experience on muscle forces and knee joint reaction forces during running. Int. J. Biomed. Eng. Technol..

[36] Molnár C., Pálya Z., Kiss R.M.J.A.S. (2021). Static Balancing Ability and Lower Body Kinematics Examination of Hungarian Folk Dancers: A Pilot Study Investigating the “Kalocsai Mars” Dance Sequence. Appl. Sci..

[37] Dominici N., Ivanenko Y.P., Cappellini G., d’Avella A., Mondì V., Cicchese M., Fabiano A., Silei T., Di Paolo A., Giannini C. (2011). Locomotor primitives in newborn babies and their development. Science.

[38] Gizzi L., Nielsen J.F., Felici F., Ivanenko Y.P., Farina D. (2011). Impulses of activation but not motor modules are preserved in the locomotion of subacute stroke patients. J. Neurophysiol..

[39] Santuz A., Ekizos A., Janshen L., Baltzopoulos V., Arampatzis A. (2017). On the methodological implications of extracting muscle synergies from human locomotion. Int. J. Neural Syst..

[40] Chvatal S.A., Ting L.H. (2013). Common muscle synergies for balance and walking. Front. Comput. Neurosci..

[41] Hug F., Turpin N.A., Couturier A., Dorel S. (2011). Consistency of muscle synergies during pedaling across different mechanical constraints. J. Neurophysiol..

[42] Robinson M.A., Vanrenterghem J., Pataky T.C. (2021). Sample size estimation for biomechanical waveforms: Current practice, recommendations and a comparison to discrete power analysis. J. Biomech..

[43] Pataky T.C. (2010). Generalized n-dimensional biomechanical field analysis using statistical parametric mapping. J. Biomech..

[44] Pataky T., Robinson M., Vanrenterghem J. (2015). Vector field statistical analysis of kinematic and force trajectories. J. Biomech..

[45] Ebig M., Lephart S.M., Burdett R.C., Miller M.C., Pincivero D.M. (1997). The effect of sudden inversion stress on EMG activity of the peroneal and tibialis anterior muscles in the chronically unstable ankle. J. Orthop. Sports Phys. Ther..

[46] Pinney S.J., Hansen S.T., Sangeorzan B.J. (2002). The effect on ankle dorsiflexion of gastrocnemius recession. Foot Ankle Int..

[47] Feger M.A., Donovan L., Hart J.M., Hertel J. (2015). Lower extremity muscle activation in patients with or without chronic ankle instability during walking. J. Athl. Train..

[48] Feger M.A., Donovan L., Hart J.M., Hertel J. (2014). Lower extremity muscle activation during functional exercises in patients with and without chronic ankle instability. PMR.

[49] Caulfield B., Crammond T., O’Sullivan A., Reynolds S., Ward T. (2004). Altered ankle-muscle activation during jump landing in participants with functional instability of the ankle joint. J. Sport. Rehabil..

[50] Wright I., Neptune R., van den Bogert A.J., Nigg B. (2000). The influence of foot positioning on ankle sprains. J. Biomech..

[51] Matsunaga N., Imai A., Kaneoka K. (2017). Comparison of muscle synergies before and after 10 minutes of running. J. Phys. Ther. Sci..

[52] Jones G.M., Watt D. (1971). Observations on the control of stepping and hopping movements in man. J. Physiol..

[53] Neptune R.R., Wright I.C., Van Den Bogert A.J. (1999). Muscle coordination and function during cutting movements. Med. Sci. Sports Exerc..

[54] Kawada M., Takeshita Y., Miyazaki T., Nakai Y., Hata K., Nakatsuji S., Kiyama R. (2020). Contribution of hip and knee muscles to lateral knee stability during gait. J. Phys. Ther. Sci..

[55] Yu P., Fernandez J. (2024). Alterations in Lower Limb Biomechanical Characteristics during the Cutting Manoeuvre in Chronic Ankle Instability Population and Copers. Phys. Act. Health.

[56] Ortiz A., Olson S.L., Etnyre B., Trudelle-Jackson E.E., Bartlett W., Venegas-Rios H.L. (2010). Fatigue effects on knee joint stability during two jump tasks in women. J. Strength. Cond. Res..

[57] Xu D., Quan W., Zhou H., Sun D., Baker J.S., Gu Y. (2022). Explaining the differences of gait patterns between high and low-mileage runners with machine learning. Sci. Rep..

[58] Xu D., Zhou H., Quan W., Gusztav F., Wang M., Baker J.S., Gu Y. (2023). Accurately and effectively predict the ACL force: Utilizing biomechanical landing pattern before and after-fatigue. Comput. Methods Programs Biomed..

[59] Xu D., Lu J., Baker J.S., Fekete G., Gu Y. (2022). Temporal kinematic and kinetics differences throughout different landing ways following volleyball spike shots. P. I Mech. Eng. P-J. SPO.

[60] Shultz S.J., Carcia C.R., Perrin D.H. (2004). Knee joint laxity affects muscle activation patterns in the healthy knee. J. Electromyogr. Kinesiol..

[61] Andrish J. (2004). The biomechanics of patellofemoral stability. J. Knee Surg..

[62] Chmielewski T.L., Rudolph K.S., Snyder-Mackler L. (2002). Development of dynamic knee stability after acute ACL injury. J. Electromyogr. Kinesiol..

[63] Lephart S.M., Ferris C.M., Riemann B.L., Myers J.B., Fu F.H. (2002). Gender differences in strength and lower extremity kinematics during landing. Clin. Orthop. Relat. Res..

